# The Neurobehavioral Effects of Buprenorphine and Meloxicam on a Blast-Induced Traumatic Brain Injury Model in the Rat

**DOI:** 10.3389/fneur.2021.746370

**Published:** 2021-10-12

**Authors:** Laura M. Anderson, Sridhar Samineni, Donna M. Wilder, Marisela Lara, Ondine Eken, Rodrigo Urioste, Joseph B. Long, Peethambaran Arun

**Affiliations:** ^1^Veterinary Services Program, Center for Enabling Capabilities, Walter Reed Army Institute of Research, Silver Spring, MD, United States; ^2^Blast-Induced Neurotrauma Branch, Center for Military Psychiatry and Neuroscience, Walter Reed Army Institute of Research, Silver Spring, MD, United States

**Keywords:** blast exposure, traumatic brain injury, neurobehavioral effects, analgesic, meloxicam, buprenorphine

## Abstract

Previous findings have indicated that pain relieving medications such as opioids and non-steroidal anti-inflammatory drugs (NSAIDs) may be neuroprotective after traumatic brain injury in rodents, but only limited studies have been performed in a blast-induced traumatic brain injury (bTBI) model. In addition, many pre-clinical TBI studies performed in rodents did not use analgesics due to the possibility of neuroprotection or other changes in cognitive, behavioral, and pathology outcomes. To examine this in a pre-clinical setting, we examined the neurobehavioral changes in rats given a single pre-blast dose of meloxicam, buprenorphine, or no pain relieving medication and exposed to tightly-coupled repeated blasts in an advanced blast simulator and evaluated neurobehavioral functions up to 28 days post-blast. A 16.7% mortality rate was recorded in the rats treated with buprenorphine, which might be attributed to the physiologically depressive side effects of buprenorphine in combination with isoflurane anesthesia and acute brain injury. Rats given buprenorphine, but not meloxicam, took more time to recover from the isoflurane anesthesia given just before blast. We found that treatment with meloxicam protected repeated blast-exposed rats from vestibulomotor dysfunctions up to day 14, but by day 28 the protective effects had receded. Both pain relieving medications seemed to promote short-term memory deficits in blast-exposed animals, whereas vehicle-treated blast-exposed animals showed only a non-significant trend toward worsening short-term memory by day 27. Open field exploratory behavior results showed that blast exposed rats treated with meloxicam engaged in significantly more locomotor activities and possibly a lesser degree of responses thought to reflect anxiety and depressive-like behaviors than any of the other groups. Rats treated with analgesics to alleviate possible pain from the blast ate more than their counterparts that were not treated with analgesics, which supports that both analgesics were effective in alleviating some of the discomfort that these rats potentially experienced post-blast injury. These results suggest that meloxicam and, to a lesser extent buprenorphine alter a variety of neurobehavioral functions in a rat bTBI model and, because of their impact on these neurobehavioral changes, may be less than ideal analgesic agents for pre-clinical studies evaluating these neurobehavioral responses after TBI.

## Introduction

Blast-induced traumatic brain injury (bTBI) in rodents is a widely used preclinical model for the injury in humans, especially deployed service members exposed to blast. Estimates of numbers of service members deployed to Iraq and Afghanistan who sustained a concussion, also called a mild traumatic brain injury (mTBI) during their deployment, is between 12 and 18% (1–3). Further, it has been estimated that up to 79% of the mTBI cases experienced by deployed service members are related to blast exposures (2–4). These injuries may or may not involve loss of consciousness or altered mental status. Single and repeated concussions in adults are significantly associated with dizziness, headache, tinnitus, cognitive deficits, and psychosocial symptoms such as depression, irritability, and PTSD (1, 3–11). Medical management of concussion is currently limited to symptomatic treatments, education, and normalization of symptoms (4, 12). Animal models of bTBI aim to define the underlying etiology in order to identify therapeutic targets and appropriate interventions to enhance overall patient recovery beyond symptomatic treatments.

Preclinical studies of bTBI in experimental rodent animal models have shown neurobehavioral and neurodegenerative abnormalities with both single and repeated blast injuries (13–23). These bTBIs have led to neuroaxonal degeneration, neuromotor dysfunctions, anxiety and depressive-like symptoms, and cognitive impairments that are both acute and delayed in nature (13–23). Blast intensity and increasing number of exposures to blast is positively correlated with the severity of these injuries (13–15) and to more chronic post-traumatic stress disorder-like symptoms in rats (24–26).

Preclinical studies of TBI in animals are often performed without using pain relieving medications, such as opioids, prompted by concerns that these medications can alter the injury-induced neurobiological perturbations and the resultant behavioral, physiologic, pathologic, and cognitive outcomes after TBI (27–29) and have been shown to be neuroprotective in some cases (30, 31). The mechanism(s) underlying opioids' neuroprotective effect remains to be elucidated, but it is hypothesized to be due to their action as inhibitory modulators of neurons (30) mediated through interactions with specific opioid receptors (31). Opioids have also been shown to have a multitude of immunomodulatory effects (32, 33) and anti-inflammatory actions (34, 35) *in vivo*, which could also account for alterations in these behavioral, physiologic, pathologic, and cognitive outcomes post-TBI. Previous preclinical studies using buprenorphine in rats undergoing some type of traumatic brain or spinal cord injury have been limited and do not always agree with one another regarding whether buprenorphine affects certain behavioral, pathologic, and/or physiologic factors. For example, one study found no significant differences in gene expression, nerve conduction, functional recovery (grid walking and beam crossing), or histopathological changes between rats receiving and those not receiving buprenorphine after spinal cord injury (36). Meanwhile, another study found significant acute gene expression changes and activation of cortical microglia and thalamic astrocytes in animals given sustained-release buprenorphine after central fluid percussion brain injury (29).

Opioids work to relieve pain by binding to one or more type of opioid receptors in the brain or spinal cord: mu (μ), kappa (κ), or delta (δ). Varying levels of activation of mu receptors (mainly in the brain) and kappa receptors (mainly in the spinal cord) by opioid drugs are the primary mechanisms of action that produce analgesia in animals (37, 38). One opioid drug in particular that is widely used in laboratory animals is buprenorphine, a partial mu receptor agonist with kappa receptor antagonist activity. Buprenorphine is approximately 25 times more potent than morphine when given intramuscularly, and lasts between 6–12 h in rats (38, 39). A newer, longer-acting formulation of buprenorphine injection (Simbadol^®^, Zoetis, Parsippany, NJ) has been studied and approved for use in cats as a 24 h pain relieving opioid (40), however there are no pharmacokinetic or scientific articles describing the use of Simbadol^®^ in experimental animals.

Non-steroidal anti-inflammatory drugs (NSAIDs) constitute another class of pain relieving medications that has been shown to alter the behavioral, physiologic, and cognitive outcomes after TBI in rodent animal models, and are therefore not often used in preclinical studies. In general, NSAIDs work by reducing arachidonic acid metabolism, which dampens the inflammatory cascade. NSAIDs interact with the cyclooxygenase (COX) enzyme, of which there are numerous forms (COX-1, COX-2, COX-3), to exert their anti-inflammatory effect. The interaction of these NSAIDs with the COX enzyme blocks the production of prostaglandin G (PGG2), an important intermediate in the inflammatory process. COX-1 enzyme is constitutive in tissues and its products regulate physiologic parameters such as platelet activity, gastrointestinal mucosal integrity, and renal function. Therefore, inhibition of COX-1 enzyme can lead to undesirable side effects such as decreased blood clotting, failure of gastric mucosal integrity, and renal compromise. In comparison, COX-2 enzyme products are inducible and their products mainly regulate the inflammatory cascade. COX-2 selective NSAIDs are currently preferred in clinical medicine for their anti-inflammatory and antipyretic effects, along with their side effect-sparing properties (37, 38).

The most widely used COX-2 selective NSAID in laboratory animal medicine is meloxicam, however many COX-2 selective NSAIDs have been studied in models of TBI in rodents, including rofecoxib, celecoxib, nimesulide, and carprofen. Previous studies have yielded conflicting conclusions regarding the neuroprotective effects of these pain-relieving drugs. For example, carprofen was shown to be neuroprotective and induce brain cell proliferation in mice after a weight drop induction of TBI (41). The COX-2 inhibitor nimesulide produced improvements in cognitive outcome more than motor outcome following diffuse traumatic brain injury in rats (42) and a related study showed that meloxicam preserves blood-brain barrier integrity and reduces brain edema, thereby promoting neuroprotection in a weight drop model of TBI in rats (43). Meloxicam has also been shown to reduce oxidative stress and exert neuroprotection in a weight drop model of spinal cord injury in rats (44). In contrast, there are studies that fail to report a neuroprotective effect of COX-2 selective medications after TBI in rodents. For example, one previous study confirmed there is an increase in COX-2 activity in the brain following weight drop in mice and that a non-selective COX inhibitor (indomethacin) induced a neuroprotective effect while preferential COX-2 inhibitors meloxicam and nimesulide failed to show the same neuroprotective effect (45). In another study, celecoxib was shown to actually worsen motor performance and had no effect on cognitive performance in rats after controlled cortical impact TBI (46). Similarly, another study concluded that rofecoxib did not have a significant protective effect on early neuronal cell death after a lateral fluid percussion brain injury in rats (47). Taken together, these reports suggest that COX-2 inhibition exerts certain neuroprotective effects after TBI in rodents, while possibly negating the neuroprotective effects of COX-2 induction following TBI in other circumstances. Of note however, none of the previous studies evaluating selective COX-2 inhibitor effects on brain injury were performed in a blast-induced model of TBI.

In the present study, our research aim was to study the effects of single pre-exposure treatment with meloxicam or a longer-acting buprenorphine formulation (Simbadol^®^) on neurobehavioral functions in rats exposed to tightly coupled repeated blasts using an advanced blast simulator. The results will help determine if these analgesics would significantly alter the results of common behavioral assays used in bTBI studies and whether their use may or may not be warranted in preclinical TBI studies in rodents. This study is not meant to be directly translational to human TBI therapies or a direct study of the efficacy of the analgesics used.

## Materials and Methods

### Animals

Forty-eight (48) male Sprague Dawley rats (Charles River Laboratories, Wilmington, MA) at 7–8 weeks old, weighing 250-275 grams were housed in individually ventilated cages at 20-22 °C on a 12:12 h light/dark cycle. Rats were provided free choice standard rat chow (Prolab IsoPro RMH3000 from LabDiet, St. Louis, MO) and chlorinated water *ad libitum* throughout the course of the study. Animal experiments were performed at the Walter Reed Army Institute of Research (AAALAC International accredited) in Silver Spring, MD under an Institutional Animal Care and Use Committee approved protocol. Rats were randomized into one of four treatment groups: sham, repeated blast (BB), repeated blast pre-treated with buprenorphine (BB+BUP), and repeated blast pre-treated with meloxicam (BB+MEL), with each group containing twelve (12) rats. The rats were pair housed before and after blast injury and participated in three different behavioral assays (rotating pole test, novel object recognition test, and open field exploration test) at multiple time points up to 28 days post-blast injury. Human observers were not blinded to treatment group for righting reflex data collection but they were blinded for all behavioral test time points. Behavioral assays and righting reflex timing data collection was performed by one of two trained individuals. For the behavioral assays, 12 animals were used per group although some animals refused to participate in the behavioral tests on certain days so the group sizes are reported as 9-12. One animal from each subgroup was used on the same day for each behavioral test. All the rats, including sham controls, underwent a skin incision on the head as these animals were also used for comparison to a group of rats that had undergone weight drop on the open skull to simulate repeated concussion for a different study purpose. The skin incisions were made twice (one week apart) and the injection of analgesics and blast exposures were carried out at day one after the second incision.

### Primary Blast Exposure

The advanced blast simulator (ABS) described previously was used for the blast exposure (22, 48). The ABS consists of a 0.5 ft long compression chamber that is separated from a 21 ft long transition/expansion test section by rupturable VALMEX^®^ membranes (Mehler technologies, VA). The compression chamber was pressurized with air, causing the membranes to rupture at a pressure dependent upon the thickness of the specific membrane sheet(s) separating the two chambers, yielding a supersonic blast wave that impacts the rats in the test section. The critical biomechanical loading to the experimental subject is determined from both the static and dynamic pressure of the blast wave, which are fully recorded by a combination of side-on and head-on piezoresistive pressure gauges (Endevco Corporation, CA) using an Astro-med TMX-18 acquisition system at a 800,000 Hz sampling rate. For blast exposure, the rats were anesthetized with 4 % isoflurane for 8 min and secured in a longitudinal (i.e. rat facing the oncoming shockwave) prone orientation in the test section of the ABS. To produce moderate injury in rats in these experiments, we used 0.034 inch thick VALMEX^®^ membranes yielding peak positive static pressures of approximately 19 psi with a positive phase duration of 4-5 msec. For tightly coupled repeated blast exposures, the rats were exposed to two 19 psi blast overpressure waves separated by 2 min as described earlier (22, 48).

For animals in the experimental groups receiving pain reducing medications, each animal was given one subcutaneous injection of either meloxicam (Metacam^®^, Boehringer Ingelheim, Duluth, GA) at 1.4 mg/kg or a longer-acting formulation of buprenorphine (Simbadol^®^, Zoetis, Parsippany, NJ) at 0.24 mg/kg based on their body weights. Body weights were taken on the day of blast injury (day 0) and again on the day of euthanasia (day 28). Both medications were selected due to their duration of action of 12–24 h of analgesic activity (40, 49, 50). The injections were given approximately 1 h prior to the blast to ensure maximum serum concentrations (40, 50, 51) and therefore maximum analgesic properties. The neurobehavioral effects of these medications on animals with repeated blast exposures were studied for a total of 28 days post-blast, when the animals were humanely euthanized. These animals were compared to repeated blast control animals that did not receive any pain relieving medications before blast and sham control animals that were anesthetized and placed in the ABS, but were not subjected to any blast overpressure exposure. Human observers were not blinded to the treatment group for righting reflex data collection, however they were blinded for all behavioral test time points. Behavioral assays and righting reflex time was performed by one of two trained individuals. Forty eight animals were used in this study, all of which were assessed for righting reflex time. For the behavioral assays, 12 animals were used per group although some animals refused to participate in the behavioral tests on certain days so the group sizes recorded are variable. One animal from each subgroup was used on the same day for each behavioral test (one sham, one BB, one BB+BUP, one BB+MEL) and the same individual animals were used for all of the behavioral evaluations until day 28.

### Rotating Pole Test

A rotating pole test was used to assess neurological motor dysfunction in the rats after bTBI, with and without pain medications (52, 53). The rotating pole test was performed as described earlier (22). The device consists of a 4 foot long wooden pole (1.5 inches in diameter) that is suspended horizontally 3 feet above the ground, overlying a foam cushion. The pole is connected to a variable speed motor at one end that rotates the pole at a constant speed. The other end of the pole is enclosed in a dark enclosed plastic box. All rats underwent training on the rotating pole prior to baseline (pre-blast) measurements. Training was performed by gently placing the rat on the pole at increasing distances from the box and clapping to encourage it to move to the box. Baseline measurements were taken one day prior to blast exposure and testing was performed on days 1, 7, 14, and 28. On each test day, each rat was given three timed trials to traverse the pole from release at the open end to passing through the opening of the black box at their own pace. They were given 120 seconds to complete each run. Once the animal entered the black box, the door was shut to allow them 30 seconds of seclusion and rest. If the animal fell (failed to traverse the pole), the time and location of the fall were recorded. Each run was scored using a protocol that incorporated balance (1 point for not falling, 0 points for falling), velocity (distance covered on the pole/time) and distance completed (1 point for a complete run, 0.75 for falling at the three-quarter mark, 0.5 for a fall at the halfway mark, and 0 points for a fall at the beginning). The two highest scores of each day were averaged as the score for that experimental subject on that day. A total of 9 to 12 rats per group were used for the rotating pole test.

### Novel Object Recognition Test

The novel object recognition (NOR) test to assess short-term memory was performed as described earlier (22). The test was performed on days 3 and 27 post-blast. There were three phases of this behavioral test. Phase 1 was the acclimation period, where rats were able to explore the empty, custom-built testing chamber (79 × 79 x 35 cm) for 5 min each day for 3 consecutive days. In the second phase, which took place the day after completion of acclimation, two identical objects (glass bottles) were placed in the chamber and the rats were allowed to explore them for 5 min total. Finally, after 20 min had elapsed from the end of phase 2, the third phase of testing began. In the third phase of testing, one of the previously explored glass bottles was replaced with a glass bottle of a different shape, and the rats were again given 5 min to explore the familiar and the novel objects in the chamber. Testing chamber settings were set to monitor the rats for time spent exploring the familiar and novel objects. Behaviors were recorded and analyzed using SMART video tracking system and software (Harvard Apparatus, Holliston, MA). A discrimination index was calculated using the formula: (Time spent with novel object - Time spent with familiar object)/Total time spent with both objects. A total of 11 to 12 rats per group were used for the NOR test.

### Open Field Test

Open field tests are often used to measure locomotor activity and anxiety-like behaviors in rodents (53–55). The rats' ambulatory behavior was measured using a locomotor activity apparatus (Omnitech Electronics, Columbus, OH). For this assessment, one animal at a time was placed in the center of the open field arena (50 × 50 × 30 cm) and allowed to freely explore the inside for one h. Activities such as horizontal activity, vertical activity, ambulatory activity, total distance moved, and time spent/distance traveled at the center and margins of the apparatus were measured using Fusion Software (Omnitech Electronics, Columbus, OH). The open field explorations were monitored on days 1, 7, 14, and 28 post-blast exposures. A total of 9 to 12 rats per group for different time-points were used for this test.

### Statistical Analysis

Statistical analysis was performed using a two-way analysis of variance (ANOVA) followed by Tukey's *post-hoc* test using multiple comparisons (GraphPad Prism 6 software). Values are expressed as mean ± standard error of the mean (SEM). When the data was non-parametric, as in the case of rotating pole test scores, a Kruskal-Wallis test was performed (GraphPad Prism 6 software). A *p* value less than 0.05 was considered significant and a *p* value less than 0.01 was considered highly significant.

## Results

### Buprenorphine Influenced Mortality in Blast Exposed Rats

Two rats out of twelve in the BB+BUP treatment group did not survive the repeated blast exposure, which corresponds to a 16.7% mortality rate in that group. No animals in the other groups died prior to the study termination.

### Animals Treated With Buprenorphine Took Longer to Right Themselves After Anesthesia

**Figure 1:**
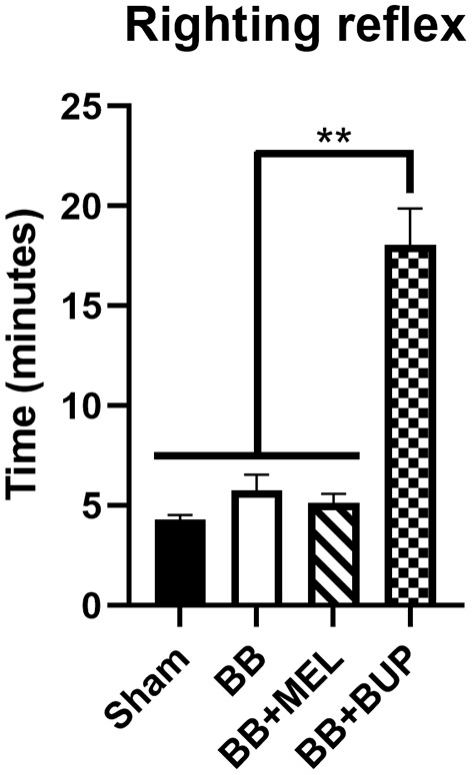
Measurement of recovery time (righting reflex time) for rats to right themselves from a supine position after isoflurane anesthesia and exit from the blast chamber. Values are expressed as mean ± standard error of the mean. Values for all four treatment groups were compared to each other (**p < 0.01, *n* = 24 rats per group).

Animals treated with buprenorphine before blast exposure (BB+BUP) took significantly longer to right themselves after isoflurane anesthesia and blast exposure than those animals with no blast exposure and not treated with pain medication (sham), those exposed to repeated blast without pain medication (BB), or those exposed to repeated blast and given meloxicam (BB+MEL) (^**^p < 0.01, Figure 1). No statistically significant changes in righting time were observed among the other groups evaluated.

### Meloxicam Showed Protection Against Vestibulomotor Deficits Induced by Blast Exposure

**Figure 2:**
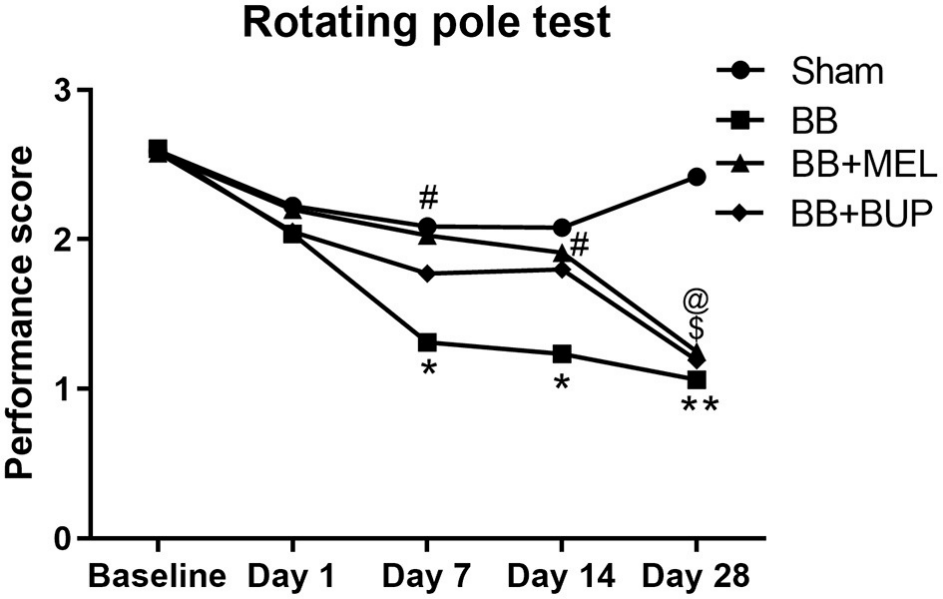
The performance of rats on the rotating pole up to 28 days post-blast exposure with and without pain medications. Values are expressed as the average of the performance scores. Performance scores of sham animals were compared to those of blast exposed animals without pain medications (*p < 0.05; **p < 0.01), treated with MEL (^$^*p* < 0.05) or treated with BUP (^@^*p* < 0.05); (*n* = 9 to 12). Performance scores of animals receiving two different pain medications were compared to those that did not receive any pain medications before blast exposure (^#^*p* < 0.05).

Repeated blast-exposed rats not given pain relieving medications showed significantly lower performance scores on days 7 and 14 (^*^p < 0.05) and highly significant deficits by day 28 (^**^p < 0.01) post-blast as compared to sham rats. Repeated blast-exposed rats treated with either meloxicam or buprenorphine did not show a statistically significant decrease in performance scores as compared to sham animals on days 1, 7, and 14; however on day 28, the decreased mean performance scores were significantly different from sham (^*^p < 0.05) (Figure 2). On days 7 and 14 post-blast, the meloxicam treated rats showed statistically significant improvements in vestibulomotor performance compared to the rats receiving no pain medication before blast (^#^*p* < 0.05). No statistically significant differences in vestibulomotor performances were observed between the groups of animals that received either meloxicam or buprenorphine before blast exposure.

### Pain Medications Promoted Short-Term Memory Deficits in Blast-Exposed Animals

**Figure 3:**
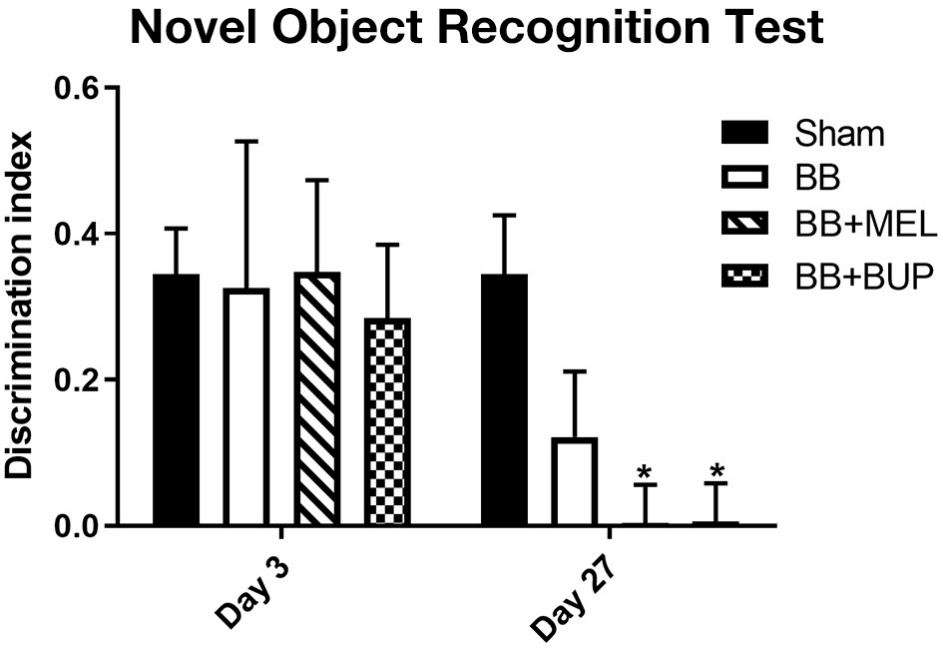
The performance of rats in the NOR test for short-term memory on days 3 and 27 post-blast exposures. The discrimination index values are expressed as mean ± standard error of the mean. The discrimination index values of sham animals were compared to those of blast exposed animals with and without pain medications (*p < 0.05, *n* = 11 to 12).

The novel object recognition test revealed no significant differences in short term memory performance in any of the groups on day 3 post-blast. The NOR test also did not show statistically significant short-term memory changes between sham and repeated blast exposed rats on day 27, although the repeated blast exposed rats showed a non-significant trend toward worsening short-term memory by day 27. In contrast, although they did not differ from vehicle-injected rats subjected to blast exposure, the NOR test did reveal statistically significant short-term memory loss on day 27 in rats given pain medications before blast exposure when compared to sham controls (^*^p < 0.05) (Figure 3).

### Pain Medications Altered Locomotor Activity and Anxiety and Depressive-Like Behaviors

#### Horizontal Activity

**Figure 4:**
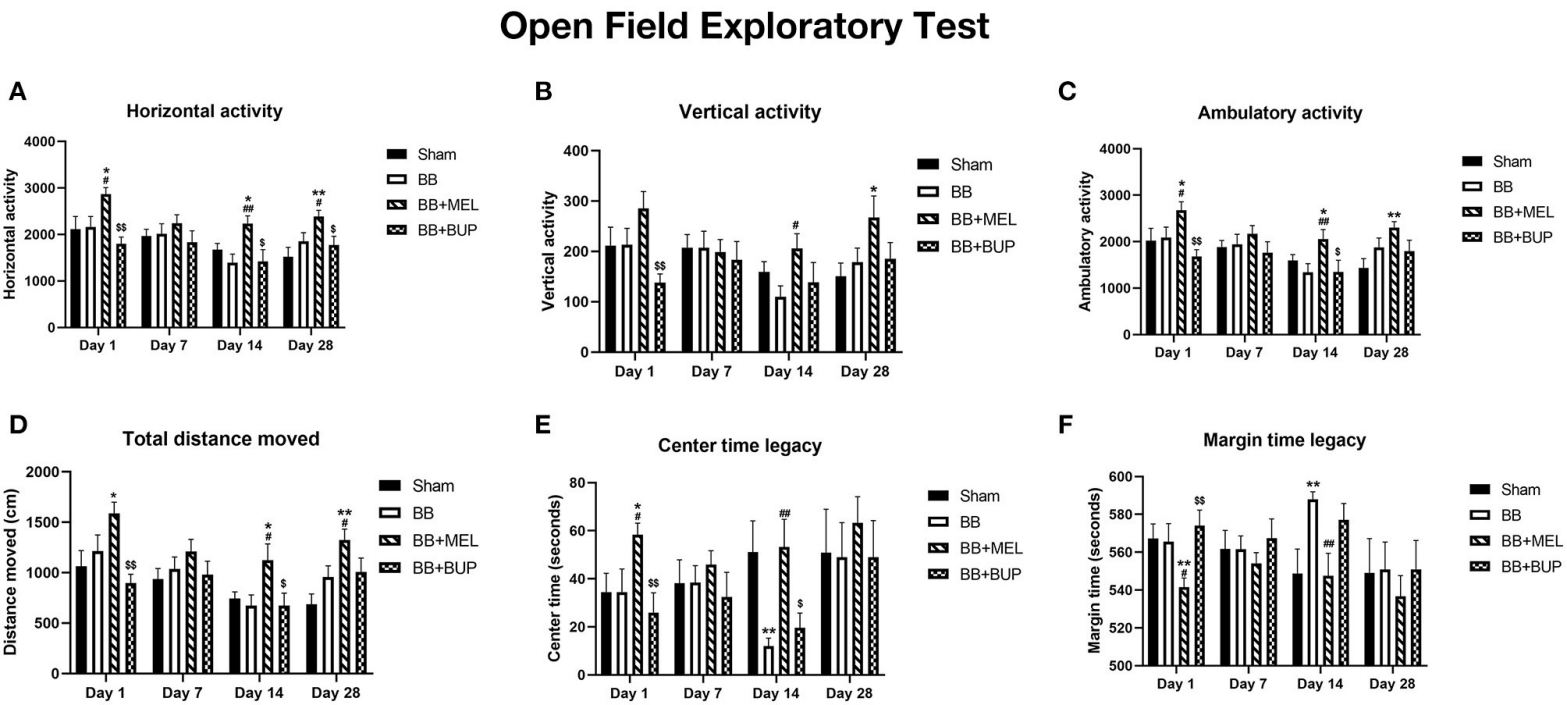
Open field exploratory activities of rats at different intervals post-blast with and without pain medications. **(A)** Horizontal activity, **(B)** Vertical activity, **(C)** Ambulatory activity, **(D)** Total distance, **(E)** Center time legacy, and **(F)** Margin time legacy during the one hr period in the open field arena. Values are expressed as mean ± standard error of the mean. Values of sham animals were compared to those of the blast exposed animals with and without pain medications (*p < 0.05, **p < 0.01). Values of blast exposed animals without pain medication were compared to the blast exposed animals receiving pain medications (#*p* < 0.05, ##*p* < 0.01). Values of animals receiving meloxicam were compared to the animals receiving buprenorphine ($*p* < 0.05, $$*p* < 0.01). A total of 9 to 12 rats per group were used for each time point.

The horizontal activity, which mostly measures the animal's motor function and exploratory activity, showed significant changes on days 1, 14, and 28 (Figure 4A). Meloxicam treated rats showed significantly higher horizontal activity on days 1, 14, and 28 compared to the sham controls and rats that did not receive any pain medication before blast exposure (^*#^*p* < 0.05, ^**##^*p* < 0.01). Compared to the rats treated with meloxicam, the rats treated with buprenorphine showed significantly less horizontal activity on days 1, 14, and 28. No significant changes in horizontal activity between any groups were observed on day 7.

#### Vertical Activity

The vertical activity, which is another measure of motor function and exploratory behavior, showed changes in the rats treated with pain medications (Figure 4B). Meloxicam treated rats showed significant increase in vertical activity compared to sham controls on day 28 and compared to untreated blast exposed rats on day 14. The vertical activities of buprenorphine treated rats were significantly less on day 1 compared to the rats treated with meloxicam. No other significant changes in vertical activities were observed between other groups on the days examined.

#### Ambulatory Activity

The ambulatory activity, which is a measure of physical activity and movement patterns, showed significant changes on days 1, 14, and 28 (Figure 4C). Significantly higher ambulatory activities were observed on days 1, 14, and 28 in the rats treated with meloxicam compared to sham controls (^*^p < 0.05, ^**^p < 0.01). The ambulatory activities of meloxicam treated rats were significantly higher on days 1 and 14 than those that did not receive pain medication (^#^*p* < 0.05, ^*##*^*p* < 0.01). Compared to meloxicam treated rats, those that received buprenorphine showed significantly lower ambulatory activities on days 1 and 14 (^$^*p* < 0.05, ^$$^*p* < 0.01).

#### Total Distance Moved

The total distance moved, which is another measure of the animal's physical activities, also showed significant changes in the animals treated with pain medications before blast and not in the animals exposed to blast without pain medications on days 1, 14, and 28 (Figure 4D). Compared to sham controls, the rats that received meloxicam before blast exposure traveled significantly more distance in the open field arena on days 1, 14, and 28, but not on day 7 (^*^p < 0.05, ^**^p < 0.01). In addition, meloxicam treated rats traveled significantly more distance on days 14 and 28 than those rats that did not receive any pain medications before blast (^#^*p* < 0.05). The rats that received buprenorphine before blast exposure traveled significantly less distance in the arena on days 1 and 14 than those rats that received meloxicam (^$^*p* < 0.05, ^$$^*p* < 0.01), but was not significantly different from the distance traveled by sham or untreated blast-exposed rats.

#### Center Time Legacy

The time spent at the center of the arena, used as an assessment of anxiety and depressive-like behaviors, also showed significant changes in the blast exposed rats and the blast exposed rats treated with pain relieving medications (Figure 4E). Rats exposed to blast without pain medication spent significantly less time around the center of the arena on day 14 (^**^p < 0.01), whereas the rats treated with meloxicam before blast exposure spent significantly more time around the center on day 1 (^*^p < 0.05) compared to sham controls. Compared to the rats that did not receive any pain medications before blast, the rats that received meloxicam before blast spent more time in the center of the arena on days 1 and 14 (^#^*p* < 0.05, ^*##*^*p* < 0.01). The rats that received buprenorphine before blast exposure spent significantly less time in the center on days 1 and 14 compared to those rats that received meloxicam (^$^*p* < 0.05, ^$$^*p* < 0.01).

#### Margin Time Legacy

The time spent at the margins of the arena are the inverse of center time and also used as an indirect assessment of anxiety and depressive-like behaviors in rodents. Significant changes were seen in the blast exposed rats and the blast exposed rats treated with pain relieving medications (Figure 4F). Rats exposed to blast without pain medication spent significantly more time around the margins of the arena on day 14, whereas the rats treated with meloxicam before blast exposure spent significantly less time around the margins on day 1 compared to sham controls (^**^p < 0.01). Compared to the rats that did not receive any pain medications before blast, the rats that received meloxicam before blast spent significantly less time around the margins of the arena on days 1 and 14 (^#^*p* < 0.05, ^*##*^*p* < 0.01). The rats that received buprenorphine before blast exposure spent significantly more time around the margins of the arena on day 1 compared to the rats that received meloxicam (^$$^*p* < 0.01).

### Animals Treated With Analgesics Gained More Weight Than Their Untreated Counterparts

**Figure 5:**
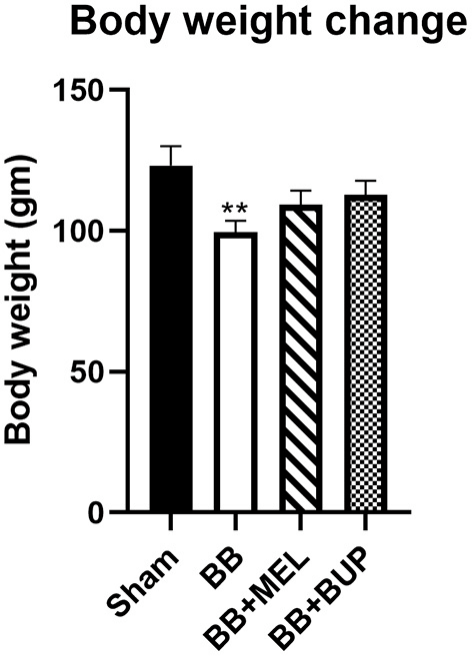
Measurement of body weight change in rats from day 0 to day 28 post-blast injury with and without pain medications. The values are expressed as mean ± standard error of the mean. Values of sham animals were compared to those of the blast exposed animals with and without pain medications (**p < 0.01). A total of 12 rats per group were used, except for 10 rats in the BB+BUP group due to mortality.

The change in body weight for each experimental group of rats can be seen in Figure 5. Blast exposed rats not treated with either analgesic had the lowest weight gain of any of the experimental groups, and was significantly different from the sham animals (^**^p < 0.01).

## Discussion

The 16.7% mortality rate in the BB+BUP group might be attributed to the physiologically depressive side effects of buprenorphine in combination with isoflurane anesthesia and an acute brain injury due to blast. The side effects of buprenorphine in humans include sedation, bradycardia, hypotension, and increased cerebrospinal fluid (CSF) pressures along with other parasympathetic nervous system changes (e.g. miosis). These side effects generally make buprenorphine contraindicated for clinical TBI cases in humans because acute brain injury also causes a spike in CSF pressure in the central nervous system (56). In addition, isoflurane anesthesia can cause bradycardia and hypotension, among other cardiovascular derangements in both human and animal patients (37, 38). When combined with acute brain injury, these cardiovascular derangements and increased CSF pressures likely contributed to the death of these animals. These mortality rates are therefore significant and should be an important consideration in the future use of buprenorphine as an analgesic in the rat bTBI model.

Buprenorphine is classified as an opioid analgesic with the known side effects of sedation, bradycardia, hypotension, and increased cerebrospinal fluid (CSF) pressures in humans. Therefore, the increased time to righting that animals in the BB+BUP group experienced was expected due to the sedative effects of the analgesic given. This increased time to righting agrees with a previous study showing longer times to extubation in animals treated with any anesthetic or sedative agent (27). In that study, animals treated with isoflurane had the shortest time to extubation as compared with rats treated with morphine, pentobarbital, or propofol, all of which showed the longest times to extubation. Since buprenorphine is an opioid similar to morphine, it is not surprising that treatment with these drugs showed similar effects on recovery time after anesthesia. Since meloxicam is not considered a sedative or anesthetic agent, it makes sense that this pain relieving medication did not show the same effect as buprenorphine.

Rats exposed to repeated blasts showed impaired vestibular motor function as compared to sham rats by day 7 on the rotating pole, which correlates well with earlier studies from our lab (22). Pretreatment with meloxicam protected the repeated blast-exposed rats from significant vestibular motor performance degradation up to day 14, but by day 28 the protective effect had receded. This suggests that the effect of a single dose of meloxicam can preserve motor performance for longer than the duration of action of the drug [12–24 hours (49, 50)]. This acute phase protective effect was attributed to the neuroprotective effects of this NSAID medication, namely, reduction of inflammatory cytokines, free radical synthesis, and altering arachidonic acid metabolism (43–57). This same protective effect is not seen with buprenorphine, which correlates with a study performed by Santiago et al. (36) that showed buprenorphine can be used for post-operative pain alleviation after spinal cord injury without affecting behavioral, physiological, or anatomical parameters in rats (36). Future studies should further characterize this protective effect, and could include measuring physiologic data, cytokine levels, interleukin and inflammatory biomarkers, and performing brain histopathology on these cohorts of animals. It would also be worthwhile to investigate whether post-blast rather than pre-blast treatment with meloxicam would be protective of acute vestibular motor deficits in these rats.

Repeated blast-exposed animals not treated with pain relievers showed a non-significant trend toward worsening short-term memory at day 27 post-blast. In addition, repeated blast-exposed animals treated with meloxicam or buprenorphine showed significant short-term memory loss at day 27 compared to sham. None of the groups showed statistically significant differences at day 3 post-blast. These findings suggest that loss of short-term memory is time-dependent and may be influenced by the acute anti-inflammatory and immune modulating effects of the pain relieving medications. Further studies are warranted to understand the pathological mechanism responsible for short-term memory deficits after TBI and how that is affected by pain relieving medications at more subacute time points.

Changes in locomotor activity and anxiety-like behaviors were observed in a biphasic pattern after repeated blast exposures with or without pain medications (Figures 4A–F). Interpretation of the open field test is based on that fact that rats are prey animals, so by nature they will preferentially remain close to vertical surfaces (i.e. walls in an open field exploratory chamber) where they would feel safer from predators. Spending more time exploring the center of an open arena would suggest a decrease in anxiety and depressive-like behaviors or caution in a rodent (58). In our study, animals treated with meloxicam showed higher levels of locomotor and exploratory activity as compared to all other groups. This could suggest that meloxicam affects the animals' overall activity level, whereas buprenorphine does not. The increased amount of time the meloxicam treated animals spent in the center rather than the margin of the open field was also significantly different from sham and repeated blast-exposed animals at a variety of time points, which may be interpreted as a reduction in anxiety and depressive-like behaviors or may be due to the overall increase in locomotor and exploratory activity seen. To investigate this change more thoroughly in future studies, additional tests of anxiety could be performed, such as the elevated plus maze. Investigating the effects of post-exposure treatment with meloxicam would be appropriate for future studies aimed at identifying therapeutic targets for human translational studies. Giving additional doses of meloxicam rather than a single dose is also warranted in future studies, as is characterizing the effects of these drugs at more chronic time-points (e.g. 6–12 months post-blast).

The explanation for why meloxicam altered the overall locomotor activity and possibly reduced the anxiety and depressive-like behaviors of these repeated blast-exposed rats is currently unknown. We postulate that it is due to the drug's mechanism of action in dampening the inflammatory cascade via COX-2 enzyme inhibition. Reducing the pro-inflammatory cells, cytokines, and chemokines produced during the acute phase of TBI has previously been postulated as neuroprotective against further delayed phase brain insult and a possible treatment modality for humans after a TBI (59–61). It stands to reason that administering meloxicam to these animals may have inhibited the secondary pathological mechanisms that often occur days to weeks after the initial insult and lead to many of the cognitive and behavioral sequelae seen. Future studies could aim to qualify and quantify these pro-neuroinflammatory and neurodegenerative biomarkers in the plasma, brain, and/or cerebrospinal fluid to further study the biochemical effects of these pain relieving drugs. In addition, future studies looking at the post-exposure efficacy of these analgesics in reducing pain in this rat bTBI model would help researchers choose the most appropriate analgesic drug for their pre-clinical bTBI rodent models.

In the current study, the repeated blast exposed animals not treated with either pain-relieving medication showed no difference in open field exploratory parameters from the sham animals at any of the acute time-points. This finding did not correlate with previous studies from our lab (22) and was attributed to the fact that the sham animals had undergone a minor skin incision the previous day for a different portion of the study. It is postulated that the animals were experiencing residual pain from this procedure during the acute time points of the behavioral assessments. This possible confounder is acknowledged as a drawback to this study, however previous studies from our lab have shown a significant decrease in locomotor activities and significant increase in anxiety-like behaviors in rats exposed to single and repeated blasts at acute time points (days 1 and 6) (22). Another acknowledged limitation of this study was the omission of sham groups treated with analgesics (sham+BUP and sham+MEL), which would have been more appropriate control groups for distinguishing the effects of the analgesic treatments from the effects of the bTBI.

Body weights of the rats are generally expected to increase over time as the animals grow, as was seen in this study. However, acute and chronic pain have been implicated in reduced feeding and weight gain in animals over time (37, 38). Our results are consistent with this phenomenon and support the concept that rats treated with analgesics to alleviate pain from the blast injury ate more than their counterparts that were not treated with analgesics. This in turn supports that both buprenorphine and meloxicam were effective in alleviating at least some of the pain these rats experienced post-blast injury.

One subcutaneous dose of the pain relieving medications meloxicam and buprenorphine, had a variety of sedative, vestibulomotor, short-term memory, and behavioral effects in rats at acute and subacute time points after tightly-coupled repeated blast exposures in an ABS. Several effects were found to change over the 28 day timeline of the study, indicating the importance of considering NSAID and opioid drug effects on mortality and neurobehavioral functions in a rat bTBI model and perhaps making them less than ideal analgesic agents during pre-clinical studies evaluating neurobehavioral changes after TBI.

## Data Availability Statement

The raw data supporting the conclusions of this article will be made available by the authors, without undue reservation.

## Ethics Statement

The animal study was reviewed and approved by IACUC, Walter Reed Army Institute of Research.

## Author Contributions

PA, LA, SS, and JL designed the experiments. DW and RU performed drug injections and blast exposures. OE, ML, and RU performed neurobehavioral tests. PA, LA, and SS analyzed the data and wrote the manuscript. All authors contributed to the article and approved the submitted version.

## Funding

This study was funded by the Military Operational Medicine Research Program at United States Army Medical Research and Materiel Command and the Center for Enabling Capabilities at Walter Reed Army Institute of Research.

## Author Disclaimer

Material has been reviewed by the Walter Reed Army Institute of Research. There is no objection to its presentation and/or publication. The opinions or assertions contained herein are the private views of the author, and are not to be construed as official, or as reflecting true views of the Department of the Army or the Department of Defense. Research was conducted under an approved animal use protocol in an AAALAC International-accredited facility in compliance with the Animal Welfare Act and other federal statutes and regulations relating to animals and experiments involving animals and adheres to principles stated in the *Guide for the Care and Use of Laboratory Animals*, NRC Publication, 2011 edition.

## Conflict of Interest

The authors declare that the research was conducted in the absence of any commercial or financial relationships that could be construed as a potential conflict of interest.

## Publisher's Note

All claims expressed in this article are solely those of the authors and do not necessarily represent those of their affiliated organizations, or those of the publisher, the editors and the reviewers. Any product that may be evaluated in this article, or claim that may be made by its manufacturer, is not guaranteed or endorsed by the publisher.
